# A Wide-Band Digital Lock-In Amplifier and Its Application in Microfluidic Impedance Measurement

**DOI:** 10.3390/s19163519

**Published:** 2019-08-11

**Authors:** Kan Huang, Yangye Geng, Xibin Zhang, Dihu Chen, Zhigang Cai, Min Wang, Zhen Zhu, Zixin Wang

**Affiliations:** 1 School of Electronics and Information Technology, Sun Yat-Sen University, Xingang Xi Road 135, Guangzhou 510275, China; 2 Key Laboratory of MEMS of Ministry of Education, School of Electronic Science and Engineering, Southeast University, Sipailou 2, Nanjing 210096, China; 3 Guangzhou Institute of Geochemistry, Chinese Academy of Sciences, Kehua Street 511, Guangzhou 510640, China

**Keywords:** digital lock-in amplifier, wide-band, fully differential analog circuit, reconfigurable hardware, electrical impedance spectroscopy

## Abstract

In this work, we report on the design of a wide-band digital lock-in amplifier (DLIA) of up to 65 MHz and its application for electrical impedance measurements in microfluidic devices. The DLIA is comprised of several dedicated technologies. First, it features a fully differential analog circuit, which includes a preamplifier with a low input noise of 4.4 nV/√Hz, a programmable-gain amplifier with a gain of 52 dB, and an anti-aliasing, fully differential low-pass filter with −76 dB stop-band attenuation. Second, the DLIA has an all-digital phase lock loop, which features a phase deviation of less than 0.02° throughout the frequency range. The phase lock loop utilizes an equally accurate period-frequency measurement, with a sub-ppm precision of frequency detection. Third, a modified clock link is implemented in the DLIA to improve the signal-to-noise ratio of the analog-to-digital converter affected by clock jitter of up to 20 dBc. A series of measurements were performed to characterize the DLIA, and the results showed an accurate performance. Additionally, impedance measurements of standard-size microparticles were performed by frequency sweep from 300 kHz to 30 MHz, using the DLIA in a microfluidic device. Different diameters of microparticle could be accurately distinguished according to the relative impedance at 2.5 MHz. The results confirm the promising applications of the DLIA in microfluidic electrical impedance measurements.

## 1. Introduction

Digital lock-in amplifiers (DLIAs) have been widely used for measuring weak signals in numerous fields [1], such as Raman spectroscopy [2], atomic force microscopy [3,4], multifunctional scanning tunneling microscopy [5], and sensors and actuators [6,7]. The measurement principle of LIAs [8] carries out a correlative demodulation with the same reference frequency as the carrier signal to single out the component of the signal at a specific reference frequency and phase.

Recently, a variety of works have been published on the implementation of high-frequency and high-precision DLIAs. Cheng et al. [9] introduced a DLIA that can reach 1 MHz measurements. It included a low-noise amplifier (LNA) and a high-speed data-acquisition–processing system-in-package (SiP). Gervasoni et al. [10] focused on high-precision and -frequency DLIA and designed a 10 MHz DLIA with sub-ppm precision. It adopted a switched ratiometric technique based on two analog-to-digital converters (ADCs) that acquired signals alternately. Zurich Instrument realized a commercial DLIA, HF2LI [11], which has a wide operation range of up to 50 MHz. Even though all the above researchers have contributed tremendously to the improvement of the operation range and measurement precision of DLIAs, a method by which to realize a wide-band DLIA is still lacking.

Wide-band DLIA could be used in impedance measurement, which has ever been implemented by complementary metal oxide semiconductor (CMOS) technology. Andrew et al. [12] implemented an electrical impedance spectroscopy (EIS) system based on CMOS technology, which had a 1 mHz to 100 kHz frequency range with a 2.65% average percentage error. Pangiotis et al. [13] developed a CMOS magnitude and phase measurement chip for impedance spectroscopy with a frequency range of 100 Hz to 100 kHz. The magnitude measurement accuracy of the chip was 98.6% ± 0.68%, and the phase measurement accuracy was 1.78° ± 0.115°. Morgan et al. [14] reported the use of microfluidic impedance cytometry (MIC) to measure the impedance of a cell, which gave an excellent coefficient of variation of 1%. To extend the frequency range and improve the precision, Reference [14] used a commercial impedance scope (HF2IS, Zurich Instrument, Zurich, Switzerland) with a transimpedance amplifier (HF2TA, Zurich Instrument, Zurich, Switzerland) to build an MIC based on the digital lock-in amplifier technique with a bandwidth of 50 MHz, but the electronic design was not illustrated. Hence, a method by which to implement a wide-band DLIA is proposed herein.

In this work, a wide-band DLIA was developed, and its key features are elaborated. These features include a 6 nV/√Hz input noise and an up to −80 dB common-mode rejection ratio (CMRR) fully differential analog circuit, a sub-ppm frequency precision and 0.02° phase deviation all-digital phase lock loop (ADPLL), and a modified low jitter (less than 3 ps) clock link for improving the signal-to-noise ratio (SNR) of the ADC by 20 dBc at most. The paper is organized as follows: Section 2 introduces the architecture and main modules of the DLIA; Section 3 presents the realization of the DLIA; Section 4 reports on the performance of the DLIA; and Section 5 describes the application of the DLIA in microfluidic electrical impedance measurement.

## 2. The Digital Lock-In Amplifier Architecture

**Figure 1:**
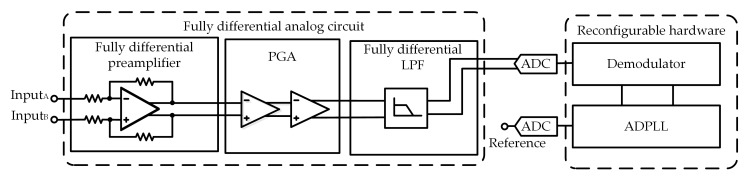
Architecture of the digital lock-in amplifier (DLIA) with two major parts: one is the fully differential analog circuit and the other is the reconfigurable hardware. (PGA is programmable gain amplifier, LPF is low-pass filter, ADC is analog-to-digital converter, ADPLL is all-digital phase lock loop.)

The architecture of the DLIA is shown in Figure 1. The analog circuit consists of a fully differential preamplifier, a programmable-gain amplifier (PGA), and a fully differential low-pass filter (LPF). The reconfigurable hardware consists of a demodulator and an ADPLL, which were implemented in an all-programmable system on chip (SoC) (XC7Z100 by Xilinx, San Jose, CA, USA).

### 2.1. Fully Differential Analog Circuit

A differential circuit has several properties that a single-ended circuit does not include. (1) It has a strong anti-interference capability because it enables the effective restraint of electromagnetic interference (EMI). (2) The propagation of a differential signal over a tightly coupled differential pair is more robust to cross-talk and discontinuity. (3) It is less sensitive to switching noise when the differential signals propagate through the connector or package [15]. With all these properties, a fully differential analog circuit for the DLIA was implemented in the following three major stages.

#### 2.1.1. The Preamplifier and Programmable-Gain Amplifier Module

The first stage of the analog circuit implementation was the preamplifier, which determines the equivalent input noise (EIN). For an N-stage amplification circuit, the EIN can be calculated as [16]:

(1) $${\mathrm{EIN}}^{2}=4\mathrm{kTR}+E_{1}{}^{2}+\frac{E_{2}{}^{2}}{A_{1}{}^{2}}+\frac{E_{3}{}^{2}}{A_{1}{}^{2}A_{2}{}^{2}}+\cdots +\frac{E_{N}{}^{2}}{A_{1}{}^{2}\ldots A_{N-1}{}^{2}}$$

where R is the impedance of the signal source, k is the Boltzmann constant, T is the Kelvin temperature, $E_{N}$ is the input noise of the n-th stage, and $A_{N}$ is the amplification gain.

Equation (1) shows that the noise of the first stage is completely added to the EIN, whereas the noise of subsequent stages is decreased by the amplification gain of the former stages. Hence, the design of the preamplifier has to consider the above features.

**Figure 2:**
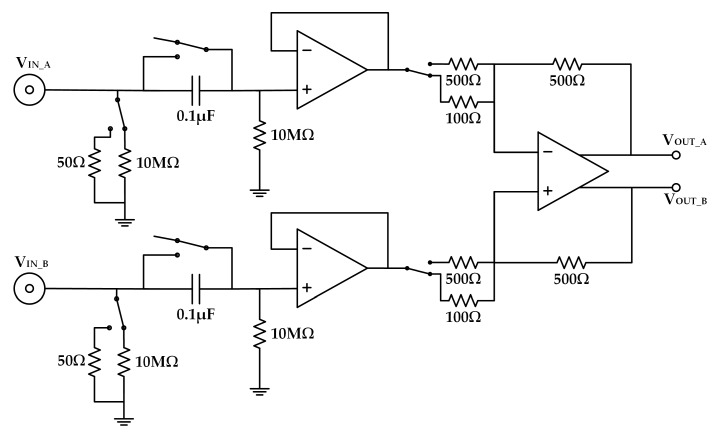
Structure of the preamplifier. The selectable 50 Ω and 10 MΩ are used for impedance matching. The selectable capacitors are for AC/DC coupling. The selectable 100 Ω and 500 Ω will change the amplification gain of the preamplifier.

Figure 2 shows that the preamplifier used consisted of three operational amplifiers (two ADA4817 by Analog Devices, Norwood, MA, USA and a LMH6552 by Texas Instruments, Dallas, TX, USA), with selectable 50 Ω and 10 MΩ resistors for impedance matching and selectable capacitors for AC/DC coupling. The operational amplifier ADA4817 had an EIN of 4 nV/√Hz at 100 kHz, a slew rate of 870 V/µs, and a unity-gain bandwidth of 1.05 GHz. The differential amplifier LMH6552 had an EIN of 1.1 nV/√Hz at 1 MHz, a slew rate of 3800 V/µs, and a unity-gain bandwidth over 1.25 GHz. The selectable 100 Ω and 500 Ω resistors for the input of LMH6552 were used for changing the amplification gain of the preamplifier. The measured EIN of the preamplifier was 4.4 nV/√Hz at 100 kHz. The input impedance was 10.08 MΩ (Supplementary Note S2). The gain–frequency response of the preamplifier is shown in Supplementary Figure S1. The increase of the 0 dB gain curve and the decrease of the 13.979 dB gain curve when the frequency exceeded 20 MHz was caused by the frequency characteristics of the LMH6552. The CMRR of the preamplifier was calculated using Equation (2), and can reach −80 dB when the gain is 13.979 dB, as depicted in Supplementary Figure S2.

(2) $$\mathrm{CMRR}=20\times \log\left(\frac{A_{d}}{A_{c}}\right)=20\times \log\left(\frac{R_{F}}{R_{G}}\times \frac{V_{com\_in}}{V_{com\_out}}\right)=20\times \log\left(\frac{R_{F}}{R_{G}}\times \frac{V_{in1}+V_{in2}}{V_{out1}+V_{out2}}\right)$$

where $A_{d}=R_{2}/R_{1}$ is the differential-mode gain, $A_{c}=V_{com\_out}/V_{com\_in}$ is the common-mode gain, $R_{F}$ is the feedback resistor, $R_{G}$ is the input resistor, $V_{in1}$ and $V_{in2}$ are the input voltage, and $V_{out1}$ and $V_{out2}$ are the output voltage.

The second stage of the analog part was the PGA module, which consisted of two cascaded differential digitally controlled VGAs (AD8370 by Analog Devices, Norwood, MA, USA). The AD8370 had a wide gain range from −11 dB to 34 dB and features of low noise, low power consumption, and high gain precision. These two stages composed the amplification unit of the DLIA, with its amplification gain as large as 65 dB.

#### 2.1.2. The Fully Differential Low-Pass Filter

**Figure 3:**
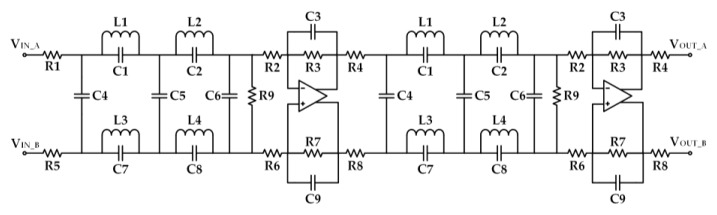
Schematic of the fully differential low-pass filter (LPF). The LPF comprised two cascading fifth-order elliptic type LC filters.

The third stage is the fully differential LPF, which is used to prevent input signals from violating the Nyquist criterion. For the high-frequency range, two cascading fifth-order elliptic type LC filters were preferred because of their simple implementation, small area consumption, and sharp cut-off characteristics. Two differential operational amplifiers (AD8139 by Analog Devices, Norwood, MA, USA) were used in the LPF. Figure 3 shows the corresponding schematic.

**Figure 4:**
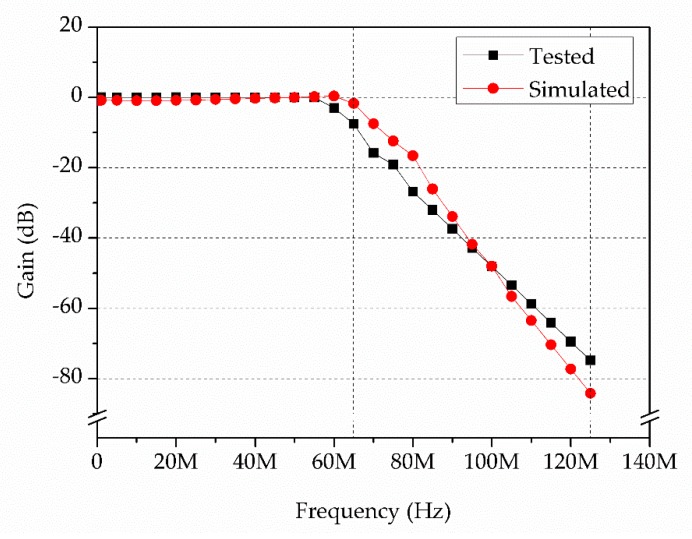
Frequency–amplitude response of the LPF.

The simulated and tested frequency–amplitude response of the LPF is shown in Figure 4. The attenuation between 65 MHz and 125 MHz in simulation was about 85 dB, whereas the board testing resulted in an attenuation of −76 dB. This difference could be attributed to the discrepancies of the passive components.

The analog circuit was implemented by such a differential structure, and the input noise of the analog circuit was 6 nV/√Hz. According to the tests, the top bound of the CMRR could reach 80 dB.

### 2.2. Reconfigurable Hardware Design

**Figure 5:**
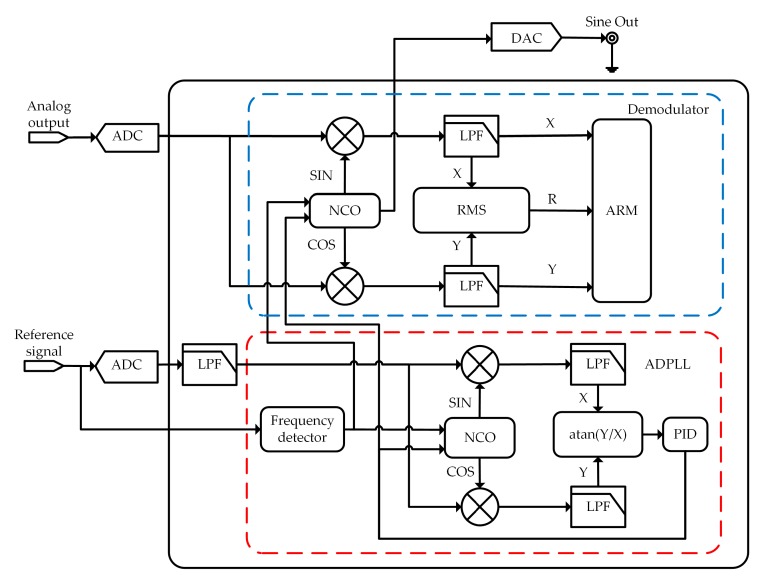
Structure of the reconfigurable hardware including a demodulator and an all-digital phase lock loop (ADPLL). (NCO is numerically controlled oscillator, RMS is root-mean-square, ARM is advanced reduced instruction set computer machine, PID is proportion-integral-differential, DAC is digital-to-noise converter.)

The reconfigurable hardware was comprised of a demodulator module and an ADPLL module, as schematically illustrated in Figure 5. The demodulator received a digitalized analog input signal through the ADC and output its amplitude and phase. The ADPLL generated a highly synchronized sine wave with the reference signal and transmitted it to the demodulator. This hardware design was implemented in the all-programmable SoC XC7Z100 from Xilinx.

#### 2.2.1. Demodulator

The demodulator consisted of a number of modules to realize the basic algorithm of the lock-in amplifier (LIA). It is essential to modulate the input signal to follow the LIA effect, whether the DLIA was in internal or external referencing mode. Hence, a numerically controlled oscillator (NCO) was integrated into the developed DLIA. It could generate synchronized reference signals, which were an in-phase signal ($S_{rx}=A_{r}\sin\left(\omega t\right)$) and an in-quadrature (90°-shifted) signal ($S_{ry}=A_{r}\cos\left(\omega t\right)$). After acquiring the input signal, the reference signals from the NCO were multiplied by the input signal in the mixers.

On the assumption that the input signal is a sine wave given by:

(3) $$S_{i}=A_{i}\sin\left(\omega t+\theta \right)+B\left(t\right)$$

where $A_{i}$, ω, and θ are the amplitude, frequency, and phase, respectively, and B(t) is the noise signal, the modulated signals were given by:

(4) $$S_{i}\times S_{rx}=\frac{1}{2}A_{i}A_{r}\cos\left(\theta \right)-\frac{1}{2}A_{i}A_{r}\cos\left(2\omega t+\theta \right)+B\left(t\right)\times A_{r}\sin\left(\omega t\right)$$

(5) $$S_{i}\times S_{ry}=\frac{1}{2}A_{i}A_{r}\sin\left(\theta \right)+\frac{1}{2}A_{i}A_{r}\sin\left(2\omega t+\theta \right)+B\left(t\right)\times A_{r}\cos\left(\omega t\right)$$

The NCO can also generate a synchronized sine signal for driving a device during a test through a digital-to-analog converter (DAC).

Since the amplitude and the phase information of the input signal were focused, the modulated signals needed to be filtered to remove the AC signals and obtain DC signals. A very narrow band low-pass filter was required to extract the DC signals, so an adaptive finite impulse response (FIR) low-pass filter (LPF) was implemented in the DLIA. These LPFs can change the roll-off characteristic from −6 dB/oct to −36 dB/oct by cascading different numbers of filters. The outputs X and Y of the LPFs are given by:

(6) $$X=\frac{1}{2}A_{i}A_{r}\cos\left(\theta \right)$$

(7) $$Y=\frac{1}{2}A_{i}A_{r}\sin\left(\theta \right)$$

In the root-mean-square (RMS) module, the RMS amplitude (R) of the input signal can be calculated by Equation (8) and then transferred to the advanced reduced instruction set computer machine (ARM) for further data processing, such as calibration by piecewise polynomial fitting (Supplementary Note S1) and data processing [17]. The phase value (θ) was also calculated in the ARM using Equation (9).

(8) $$\theta R=A_{i}/\sqrt{2}=\frac{2\sqrt{X^{2}+Y^{2}}}{A_{r}\times \sqrt{2}}$$

(9) θ=arctan(Y/X)

#### 2.2.2. All-Digital Phase Lock Loop

For high-precision detection in the external referencing mode, the precision and stability of the reference signal generated by the ADPLL determines the accuracy of further calculation of outputs *R* and *θ*. Compared with an analog phase lock loop (PLL), an ADPLL has the advantages of the absence of thermal drift, better suppression of harmonics and interference, and flexible options for data processing [18,19]. Hence, an ADPLL was preferred in our design. The ADPLL consisted of a frequency detector, a NCO, two mixers, two LPFs, an arc tangent function, and a proportion-integral-differential (PID) controller, as shown in Figure 5.

The dedicated frequency detector in our ADPLL was able to measure reference frequency *ω* with a precision of 0.38 ppm throughout the entire working range. This feature ensured that signals generated by the NCO had the same frequency as the reference signal. Conventional period measurement counts the number of rising edges of the standard signal in numerous periods of the testing signal, and then calculates the period of the testing signal [20]. However, the conventional measurement generates a large error in the high-frequency range. Therefore, frequency division is required to improve the precision of frequency measurement. Compared with the conventional period measurement, the frequency detector used here was optimized by means of the equally accurate period-frequency measurement with hysteresis. First, a measurement was conducted to estimate the frequency for selecting the appropriate division factor. Afterwards, the high frequency was divided into lower scopes in order to maintain the maximum counting error. To make the division more stable, a hysteresis operation was performed.

**Figure 6:**
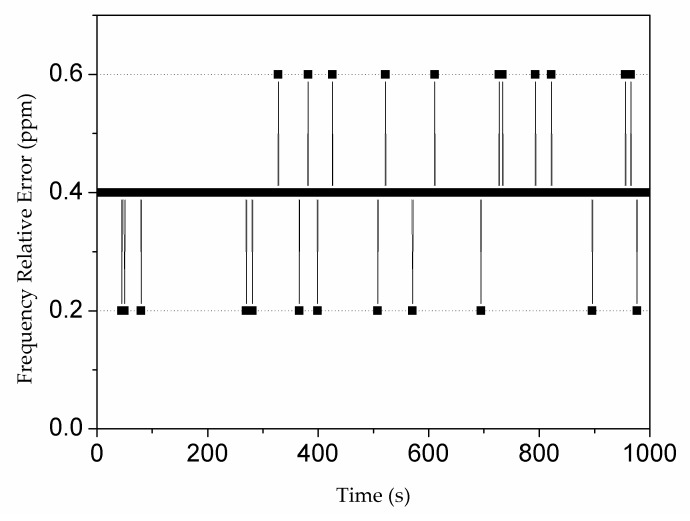
Frequency relative error for 10 MHz (a measurement of 1000 s).

In order to characterize the frequency precision, a 100 mVrms sine wave generated by a signal generator, Keysight 33622A, was transmitted to the DLIA. A series of frequencies, i.e., 1 Hz, 10 Hz, 100 Hz, …, 1 MHz, 2 MHz, …, 64 MHz, and 65 MHz, as well as some random frequencies, were applied. The ratio of measured frequency deviation to the standard frequency of the Keysight 33622A was then calculated and expressed in ppm. The measured frequency precision of 10 MHz is exemplarily displayed in Figure 6. The measured frequency precision of the 65 MHz DLIA was 0.38 ppm on average.

The LPFs after mixers were designed by the Filter Designer & Analysis Tool [21]. Here, a 250 MSPS sampling rate and 10 kHz cut-off frequency FIR filter was preferred, due to its stability and linearity.

Similar to the principle of the demodulator, the NCO in the ADPLL received frequency ω from the frequency detector and generated signals sin(ωt+θ) and cos(ωt+θ), where θ is the phase difference between the reference signal and the generated signals. Concurrently, the digitalized reference signal from the ADC passed through a LPF, which extracted the fundamental sine wave from the rectangle input reference signal, and then the filtered signal was fed back to the mixers. After the LPF filtering behind the mixers, the phase difference can be calculated by the arc tangent function:

(10) $$\theta =\arctan\left(\frac{Y}{X}\right)=\arctan\left(\frac{\frac{1}{2}\;A_{ref}\sin\left(\theta \right)}{\frac{1}{2}\;A_{ref}\cos\left(\theta \right)}\right)$$

After the phase difference was detected, the PID controller was used to adjust the output phase of the NCO to lock in the phase of the reference signal. Due to the use of PID controller, the ADPLL was able to lock in the phase of the reference signal with a deviation less than 0.02°.

#### 2.2.3. Modified Clock Link

In high-frequency applications, the clock jitter caused by the clock link usually downgrades the SNR of ADCs significantly. The effect of clock jitter on an ideal ADC SNR can be predicted by the following analysis [22]. Given an input signal $V\left(t\right)=V_{0}\sin2\pi ft$, the changing rate of this input is:

(11) $$\frac{dV}{dt}{|}_{rms}=2\pi fV_{O}\cos2\pi ft{|}_{rms}=2\pi fV_{0}/\sqrt{2}$$

Root-mean-square (RMS) voltage error $\Delta V_{rms}$ is:

(12) $$\Delta V_{rms}=2\pi fV_{0}t_{j}/\sqrt{2}$$

where $t_{j}$ is the RMS sampling clock jitter, f is the frequency of input sine signal, and $V_{0}$ is the amplitude of the input signal. The RMS value of the full-scale input sine wave is $V_{0}/\sqrt{2}$. Therefore, the RMS SNR is given by:

(13) $$\mathrm{SNR}=20log_{10}\left[\left(V_{0}/\sqrt{2}\right)/\Delta V_{rms}\right]=-20log_{10}\left(2\pi ft_{j}\right)$$

Equation (13) assumes an infinite resolution for an ideal ADC, in which the clock jitter is the only factor determining the SNR. Moreover, its theoretical maximum value decreases if the ADC has a finite resolution or when other factors are considered. Thus, the relationship between the SNR of a realistic ADC and clock jitter is given as [23]:

(14) $$\mathrm{SNR}=-20log_{10}\sqrt{{\left(2\pi ft_{j}\right)}^{2}+\frac{2}{3}{\left[\left(1+\varepsilon \right)/2^{N}\right]}^{2}+{\left[2\sqrt{2}V_{NOISE_{rms}}/2^{N}\right]}^{2}}$$

Equation (14) indicates that the SNR of a realistic ADC depends not only on the quantization noise, but also on the average differential non-linearity (DNL) error ε, clock jitter $t_{j}$, working frequency f, effective input noise $V_{NOISE_{rms}}$, and number of bits of resolution N. As such, Equations (13) and (14) exhibit that the clock jitter affects the SNR of the ADC significantly.

**Figure 7:**
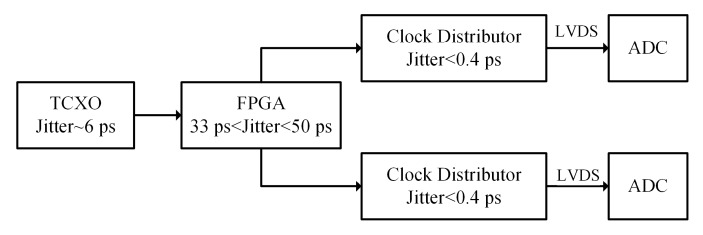
Conventional clock link. (TCXO is temperature compensation crystal oscillator, FPGA is field programable gate array, LVDS is low-voltage differential signaling.).

Figure 7 shows the SNR of an ADC driven by a conventional clock link. A 40 MHz temperature-compensation crystal oscillator (TCXO) with 6 ps clock jitter was used as the clock input of the field programmable gate array (FPGA). A 250 MHz clock signal was generated by the PLL of the FPGA and transmitted to two clock distributors. This configuration may cause the ADC a clock jitter of more than 39 ps, mainly due to the clock jitter of the FPGA [24].

**Figure 8:**
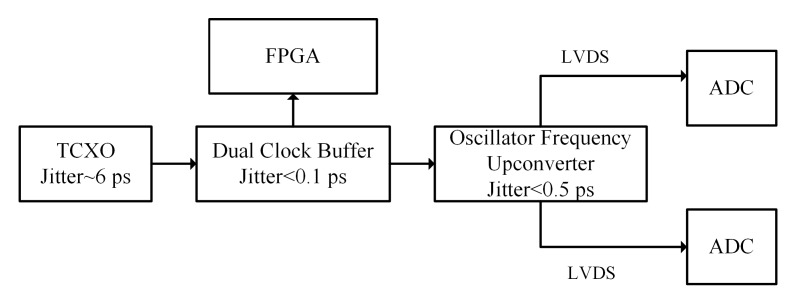
Modified clock link.

**Figure 9:**
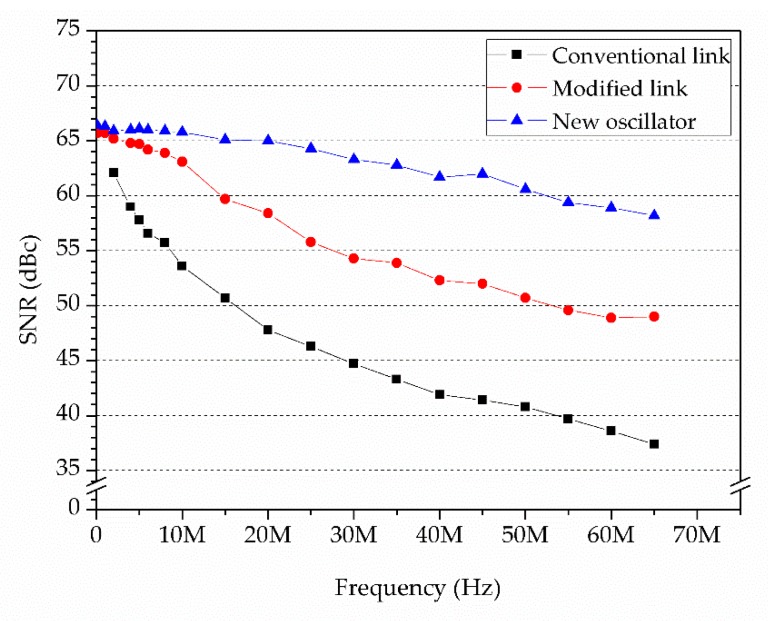
Signal-to-noise ratio (SNR) of the ADC.

To improve the SNR of the ADC, we have proposed a modified clock link (Figure 8). An oscillator frequency up-converter (AD9550 by Analog Devices, Norwood, MA, USA), which obtains the clock signal from the TCXO through a clock buffer, was used to drive the ADCs. This configuration theoretically limits the maximum clock jitter to less than 7 ps. However, practical tests yielded a value of 8.2 ps, which was mainly caused by the TXCO. To further improve the SNR of the ADC, a lower jitter TXCO was chosen. The theoretical clock jitter of the new TXCO (LFTCXO063712 by IQD Frequency Products, Somerset, UK) [25] was 2.73 ps, which led to a clock jitter of less than 3 ps for the modified clock link. In order to prove the improvement, the SNR of the ADC was measured under the above-mentioned three conditions, i.e., the conventional clock link, the modified clock link, and the clock link with a new oscillator (Figure 9). The measured SNR decreased from 62.1 dBc to 37.4 dBc as the testing frequency increased from 2 MHz to 65 MHz, which was coincident with the relationship between the clock jitter and SNR of the ADC. The result showed that the modified clock link with new oscillator could give a maximum improvement of 20 dBc of the SNR.

## 3. The DLIA Realization and Characteristics

**Figure 10:**
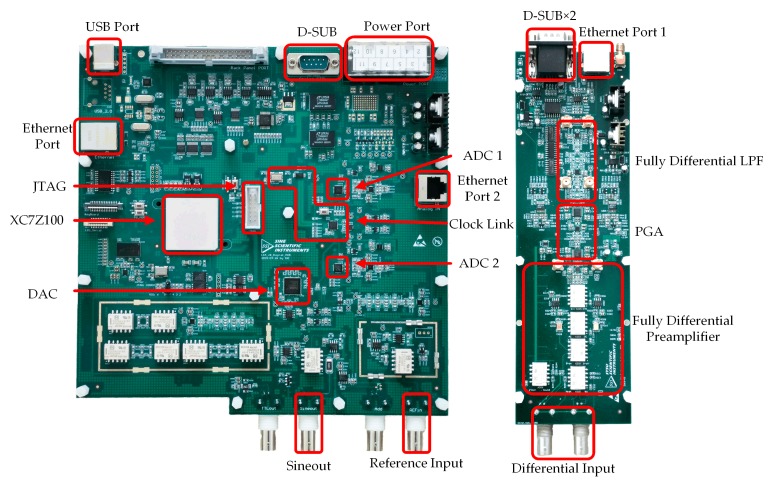
Photograph of the realized DLIA.

Figure 10 shows a prototype of the DLIA, comprising an eight-layer, 21 × 21 cm digital printed circuit board (PCB) (left in Figure 10) and a six-layer, 23.5 × 8.4 cm analog PCB (right in Figure 10).

The analog PCB consisted of a fully differential pre-amplifier (two ADA4817 by Analog Devices and a LMH6552 by Texas Instruments), PGAs (AD8370 by Analog Devices), and an anti-aliasing LPF (AD8139 by Analog Devices), as previously introduced in Section 2. It was powered by ± 8 V and further regulated to ± 5 V by its onboard low dropout regulator (LDO) (UA7905, UA7908, UA7805, and UA7808 by Texas Instruments, Dallas, TX, USA). Three pairs of sub-miniature version A (SMA) connectors were used for the differential tests of the preamplifier, PGA, and LPF, individually from bottom to top. An Ethernet port 1 at the top edge of the board conveyed the differential output of the LPF and was connected to the analog input (port 2) of the digital board. Two D-SUB connectors at the top left were the power and control input. The SMA at the top right was used as a monitor of the output of the analog board. The entire board was installed in a shielding box to prevent EMI from the external environment.

The digital board used XC7Z100 to implement the digital algorithm. Two 14 bit, 250 million samples per second (MSPS) ADCs (AD9642 by Analog Devices, Norwood, MA, USA) were used to digitalize the analog signals from the analog board and reference input. A 16 bit, 1 gigabit samples per second (GSPS) DAC (AD9779 by Analog Devices, Norwood, MA, USA) was placed for the output of a synchronized sine wave through the bottom left bayonet nut connector (BNC). Here, the reference input was connected through the lower right BNC. In order to avoid same-frequency interference, the analog input (Ethernet port 2) was intentionally placed away from the reference input and the sine wave output [26]. The USB port at the top left enabled the connection of the digital board to a PC running a LabVIEW user interface for parameter setting and signal visualization. Additionally, an Ethernet port on the top left was also integrated onto the board for data transformation. A D-SUB connector was used to control the analog board. The digital board was powered by ± 8 V and 5 V.

The characteristics of the DLIA are shown in Table 1. The proposed DLIA had a wider bandwidth of 65 MHz and a lower power consumption of 35 W. The dynamic reserve was the same as other LIAs. The input voltage noise of the proposed DLIA was 6 nV/√Hz. The sizes of the LIAs were similar.

## 4. Performance of the Digital Lock-in Amplifier

### 4.1. Measurement Settings

In order to verify the performance of the DLIA, a signal generator (Keysight 33622A) and an attenuator (Keysight 8496A) were used to generate the input signal, and the Sync signal of the Keysight 33622A was used as the reference signal. The DLIA was set in external reference mode with 50 Ω input impedance, 24 dB/oct filter roll-off, 300 ms time constant, and AC coupling.

### 4.2. Deviation and Stability of Phase Detection

**Figure 11:**
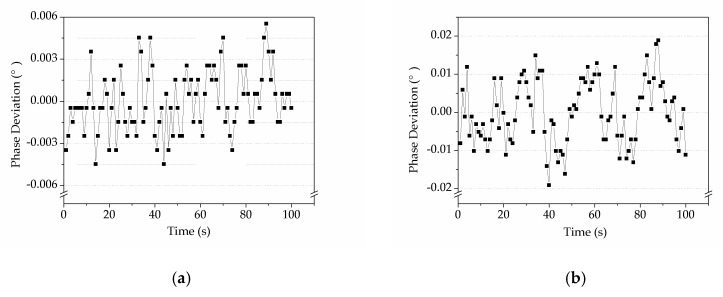
Deviation of the phase detection. (**a**) The phase detection of 100 s measurements for the 10 MHz sine wave was less than 0.006°. (**b**) The phase detection of 100 s measurements for the 50 MHz sine wave was less than 0.02°.

In order to measure the deviation and stability of the phase detection, a 100 mVrms sine wave was fed into both the signal input and reference input of our DLIA. The frequency changed from 10 Hz to 65 MHz. The sensitivity of the DLIA was set to 200 mV. The data were saved every 0.1 s for 3 h. The phase deviation was calculated as:

(15) $$\mathrm{Phase}\;\mathrm{Deviation}=P-\bar{P}$$

where P is the measured phase value and $\bar{P}$ is its average value. Figure 11 shows that the phase deviation of the DLIA was less than 0.006° and 0.02° for the 10 MHz and 50 MHz cases, respectively. In addition, the standard deviation of the phase was 0.0021° and 0.0083° for 10 MHz and 50 MHz, respectively, which verified the high stability of the DLIA. Test results of other frequencies also yielded good performance of the DLIA.

### 4.3. Amplitude–Frequency Response of the DLIA

**Figure 12:**
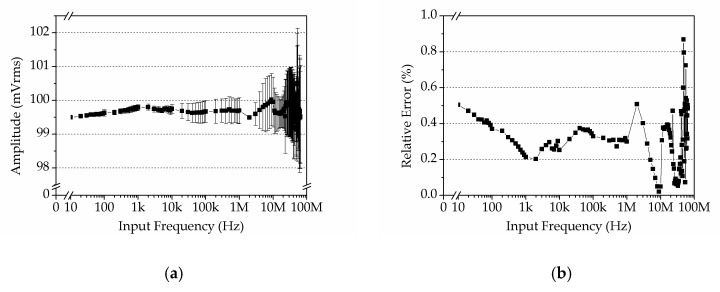
Measurement results. (**a**) Amplitude–frequency response of the DLIA. (**b**) Relative error of measurement results was less than 0.9%. The sharp change of the relative error when input frequency exceeded 10 MHz was because the parasitic effect became much effective.

In addition, the amplitude–frequency response of the DLIA was tested. A 100 mVrms sine wave was used as the input, and the frequency was swept from 10 Hz to 65 MHz. The DLIA used the same settings as above. Testing results are shown in Figure 12, where each value is an average value of 10 tests (1 test per second for 10 s).

Since the input signal was set to 100 mVrms, the result in Figure 12a demonstrates that the DLIA yielded very close values to the setting value over the entire operating range. We also changed the amplitude from 2 mVrms to 500 mVrms under the same testing settings, and found that the performance of the DLIA was similar to that at 100 mVrms. Relative error $R_{E}$ was calculated by Equation 16 and is shown in Figure 12b.

(16) $$R_{E}=\frac{\left|R_{i}-R_{T}\right|}{R_{T}}\times 100\%$$

where $R_{i}$ is the tested value and $R_{T}$ is the setting value. In the entire operating range, our DLIA could detect a signal with a relative error less than 0.9%. When the input amplitude was changed from 2 mVrms to 500 mVrms, our DLIA showed a small relative error in the entire testing range. This result validates that the DLIA can detect signal amplitude with a reasonable precision.

### 4.4. Ability to Detect Weak Signals

**Figure 13:**
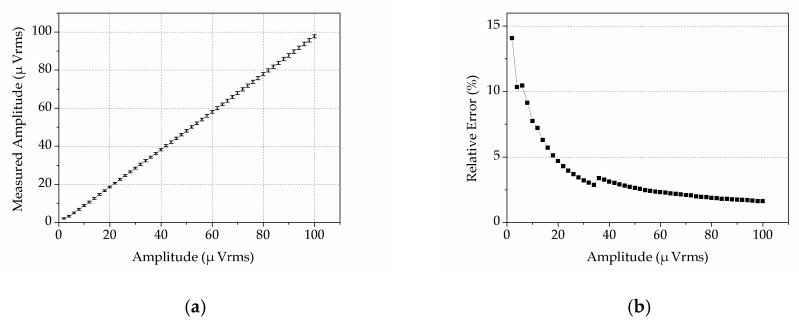
Weak signal detection using the DLIA. (**a**) Measured amplitudes versus the setting values. (**b**) Relative error of the measured amplitude. The two drops were caused by the amplification gain change.

Moreover, we characterized the ability of DLIA to detect a weak signal. Amplitude of a 10 MHz input sine wave was changed from 2 µVrms to 100 µVrms, and the sensitivity of the DLIA was changed correspondingly. The measured amplitude and relative error are shown in Figure 13. Every value is the average value of 10 tests (1 test per second for 10 s). The result proves that the DLIA was able detect weak signals from 2 µVrms to 100 µVrms, very close to the setting values (Figure 13a). The relative error of DLIA was less than 15%, and a higher input signal resulted in a smaller relative error (Figure 13b).

### 4.5. Ability to Extract Weak Signals from Background Noise

**Figure 14:**
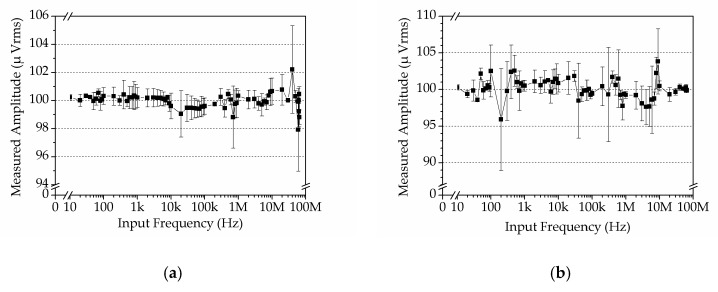
Amplitude–frequency response. (**a**) High SNR measurement. The input signal was a 100 µVrms sine wave without white noise. (**b**) Low SNR measurement. The input signal contained a 100 µVrms sine wave with 10 mV white noise.

In addition, we performed two sets of characterization to test the ability of DLIA to extract weak signals from background noise. One had a high SNR input signal, and the other one had a low SNR input signal. The input signal was generated by the Keysight 33622A and the white noise was generated by the RIGOL DG4162. The DLIA settings were the same as before. The testing frequency of the sine wave was 10 MHz, and the amplitudes were 100 µVrms with and without 10 mVrms white noise. All values in Figure 14 are the average values of 10 tests (1 test per second for 10 s). 

**Figure 15:**
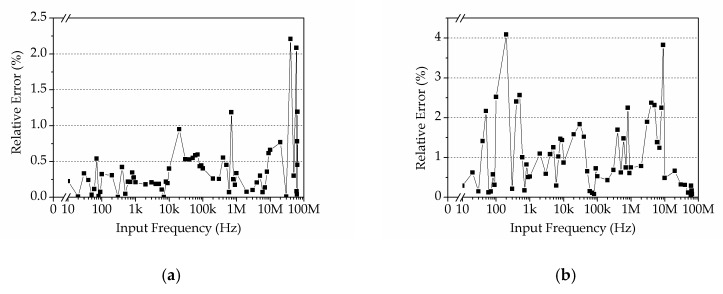
Relative error for the high SNR test (**a**) and low SNR test (**b**).

The results in Figure 14 demonstrate that the DLIA could extract weak signals with less than 5% deviation, whether the input had a high or low SNR. Moreover, the relative error at a high SNR was mostly less than 1%, except for a few points at around 60 MHz (Figure 15a). For a low SNR, the relative error was less than 4.5% (Figure 15b). Therefore, the results indicate that the DLIA can detect weak signals from background noise with a small error in the entire operating range.

## 5. Application of the DLIA in Microfluidic Electrical Impedance Measurement

### 5.1. Electrical Impedance Measurement of Single Beads in a Microfluidic Device

**Figure 16:**
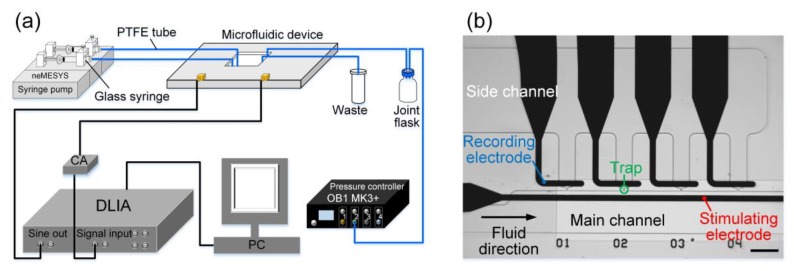
Experimental setup of electrical impedance measurement (**a**) and micrograph of the microfluidic device (**b**). Scale bar is 100 μm.

The experimental setup and the working principle of electrical impedance measurement in the microfluidic device were similar to the previous study [27]. As shown in Figure 16, two glass syringes were mounted in a precision syringe pump (neMESYS, cetoni GmbH, Korbussen, Germany) for the perfusion of medium and bead suspension into the main channel of the microfluidic device, with a constant flow rate of 0.5 μL/min. Due to laminar flow, medium and bead suspension can flow in the main channel without mutual diffusion. In order to capture beads in the traps, an underpressure generated by a pressure controller (OB1 MK3+, Elveflow, Paris, France), was applied to the side channel via polytetrafluoroethylene (PTFE) tube. By precisely regulating the fluid profile via the applied pressure at the side channel, single beads could be captured at each trap. A frequency-sweep sine signal (1 V_pp_, 300 kHz~30 MHz) from the sine out port of the DLIA was applied to the stimulus electrode. Correspondingly, the response current from the recording electrode was transformed to a voltage signal by a custom current amplifier (CA) (Supplementary Figures S4 and S5) and collected by the wide-band DLIA. 

In particular, only four traps were designed for the validation of the applications of DLIA in wide-band EIS. The throughput of the microfluidic device can be adjusted in different applications. In addition, all EIS measurements of beads were performed at the second trap, as indicated in Figure 16b.

### 5.2. Classification of Bead Diameters through Electrical Impedance Measurement Using the DLIA

Before the DLIA was used in the electrical impedance spectroscopy (EIS), we measured a 10 kΩ resistor and a 100 nF capacitor as simulations and compared with HF2LI, as shown in Supplementary Figures S6 and S7. The simulations results showed that the DLIA can measure the impedance of the resistor and capacitor correctly and with a very small standard deviation.

**Figure 17:**
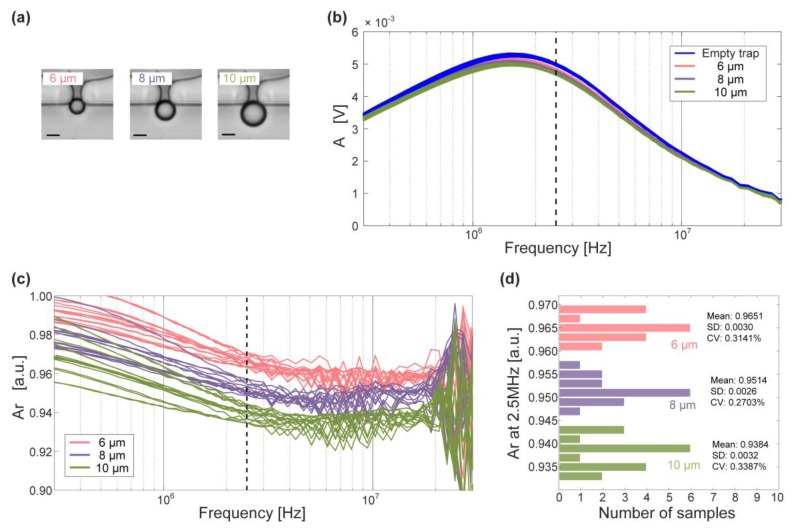
Electrical impedance measurement of beads in the microfluidic device. (**a**) Micrographs of immobilized beads with different diameters. Scale bar is 5 μm. (**b**) Raw amplitude of electrical impedance spectroscopy (EIS) signals over the swept frequency from 300 kHz to 30 MHz. (**c**) Relative amplitude of EIS signals over the swept frequency from 300 kHz to 30 MHz. (**d**) Histogram of relative amplitude of measuring 6 μm, 8 μm, and 10 μm beads at 2.5 MHz.

In order to verify the applications of DLIA in wide-band EIS, polystyrene beads (Sigma Aldrich Co., Darmstadt, Germany) with diameters of 6 μm, 8 μm, and 10 μm were measured. Single beads with standard sizes were immobilized at one trap through hydrodynamic forces (Figure 17a), and the impedance measurements were performed before and after bead immobilization. Amplitudes of measured EIS signals were normalized as:

(17) $$A_{r}=A/A_{e}$$

where *A* and *A_e_* are the amplitudes of the trap with and without immobilized beads, respectively. Furthermore, the histogram of normalized amplitudes of impedance signals at 2.5 MHz was plotted for better elucidation of impedance measurements. Additionally, the mean value, the standard deviation (SD), and the coefficient of variation (CV) of the normalized signals were calculated to evaluate the accuracy of distinguishing different beads using our DLIA in a microfluidic device.

As illustrated in Figure 17b,c, the wide-band amplitude spectra of EIS signals were obtained using the DLIA. EIS signals can reveal various information related to different ranges of swept frequency. At mid-frequencies, the amplitudes of EIS signals can reveal the size of measured samples.

As Figure 17d shows, mean values of the relative amplitude of EIS signals at 2.5 MHz had remarkable differences. Small values of SDs and CVs confirmed the stability of impedance measurements and ensured high sensitivity of measuring impedance of the immobilized samples. The three sets of beads were successfully classified using our DLIA, thereby verifying the feasibility of application of the wide-band DLIA in microfluidic EIS measurements.

Moreover, we compared the precision of bead classification using the DLIA and HF2IS (Supplementary Table S1). The differences of mean *A_r_* between 8 μm and 10 μm beads were comparable with previous study (DLIA: 0.013; HF2IS: 0.019). Additionally, the DLIA was comparable with the HF2IS in terms of the SDs and CVs. The similar results confirm that the DLIA can be used in microfluidic impedance measurements with a high precision. Based on its good bead classification performance, the DLIA is expected to be able to obtain various information (e.g., size, state) of cells, tissues, and model organisms over a wide-band frequency range in future studies. Additionally, we may integrate the microfluidic device with the DLIA by optimizing the design and the implementation to provide a portable EIS system for bio-impedance applications.

## 6. Conclusions

In this paper, the design of a wide-band DLIA has been presented. A fully differential analog circuit was implemented. The key features of design of the ADPLL have been elaborated. The ADPLL achieved a 0.38 ppm frequency detection and 0.02° phase deviation. In addition, a modified low jitter clock link was implemented to successfully increase the SNR of the ADC by 20 dBc, compared to the conventional clock link. Test results showed good performance of the DLIA, with less than 1% relative error for millivolt signal detection and 15% relative error for microvolt signal detection. The proposed DLIA has 65 MHz bandwidth, 120 dB dynamic reserve, 6 nV/√Hz input voltage noise, and 35 W power consumption. Additionally, the successful discrimination of standard-size beads with different diameters has suggested the promising applications of the DLIA in microfluidic electrical impedance measurements. In conclusion, all the experimental results demonstrated that the DLIA is capable of measuring weak signals in wide-band ranges, and can be applied to EIS measurements in microfluidic devices. In the future, the wide-band DLIA can be optimized for a smaller size and lower power consumption to make the EIS system portable for bio-impedance applications.

## Figures and Tables

**Table 1:** The Characteristics of the proposed DLIA and other LIAs.

Parameter	This Work	HF2LI	SR865A
Bandwidth(Hz)	65 M	50 M	4 M
Dynamic Reserve(dB)	120 dB	120 dB	120 dB
A/D Conversion	14 bit, 250 MS/s	14 bit, 210 MS/s	16 bit, 10 MHz
Input Voltage Noise	6 nV/√Hz	5 nV/√Hz	2.5 nV/√Hz
Size(cm in Width-Height-Depth (WHD))	46 × 45 × 13	45 × 28 × 9	43 × 43 × 13
Power	35 W	45 W	60 W

## Supplementary Materials

The following are available online at https://www.mdpi.com/1424-8220/19/16/3519/s1, Figure S1: The gain-frequency response of the preamplifier, Figure S2: The CMRR-frequency response of the preamplifier. Figure S3: The connection model of the input impedance measurement. Figure S4: The structure of the CA. Figure S5: The frequency characteristics of the CA. Figure S6: The measurement results of a 10 kΩ resistor by our DLIA and HF2LI. Figure S7: The measurement results of a 100 nF capacitor by our DLIA and HF2LI. Figure S8: The amplitude-frequency response of the HF2LI. Table S1: The comparison of the microfluidic impedance measurement between the DLIA and HF2IS. Note S1: The calibration process in our DLIA. Note S2: The input impedance measurement.
