# Supplementary material for: A Wide-Band Digital Lock-In Amplifier and Its Application in Microfluidic Impedance Measurement
Source: Sensors (Basel). 2019 Aug 11;19(16):3519. doi: 10.3390/s19163519 (PMC6719078; doi:10.3390/s19163519)
Supplement: Supplementary file 1 [file sensors-19-03519-s001.pdf]

Supporting Information

A Wide-Band Digital Lock-In Amplifier and Its Application in Microfluidic Impedance Measurement

Kan Huang [1], Yangye Geng [2], Xibin Zhang [1], Dihu Chen [1], Zhigang Cai [1], Min Wang [3], Zhen Zhu [2,*], and Zixin Wang [1,*]

[1] School of Electronics and Information Technology, Sun Yat-Sen University, Xingang Xi Road 135, Guangzhou 510275, China
[2] Key Laboratory of MEMS of Ministry of Education, School of Electronic Science and Engineering, Southeast University, Sipailou 2, Nanjing 210096, China
[3] Guangzhou Institute of Geochemistry, Chinese Academy of Sciences, Kehua Street 511, Guangzhou 510640, China
* Correspondence: wangzix@mail.sysu.edu.cn (Z.W.); zhuzhen@seu.edu.cn (Z.Z.)

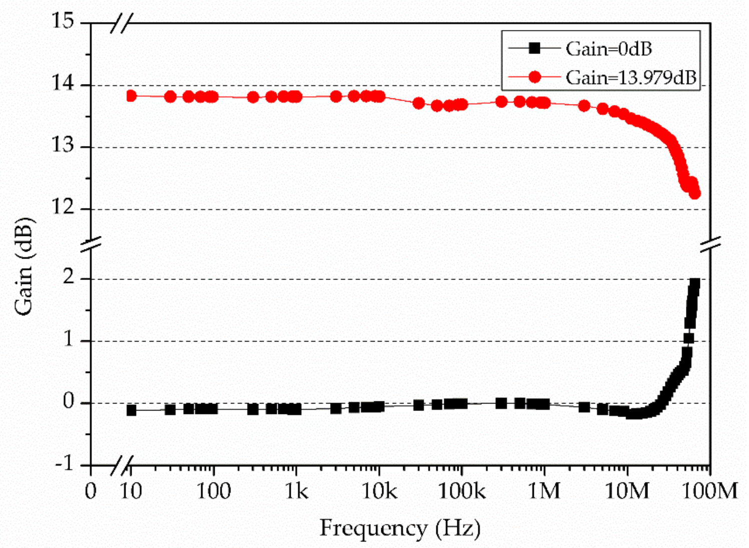

Supplementary Figure S1. The gain-frequency response of the preamplifier. The increase of the 0 dB gain and the decrease of the 13.979 dB gain when the frequency exceeds 20 MHz is caused by the frequency characteristics of the operational amplifier.

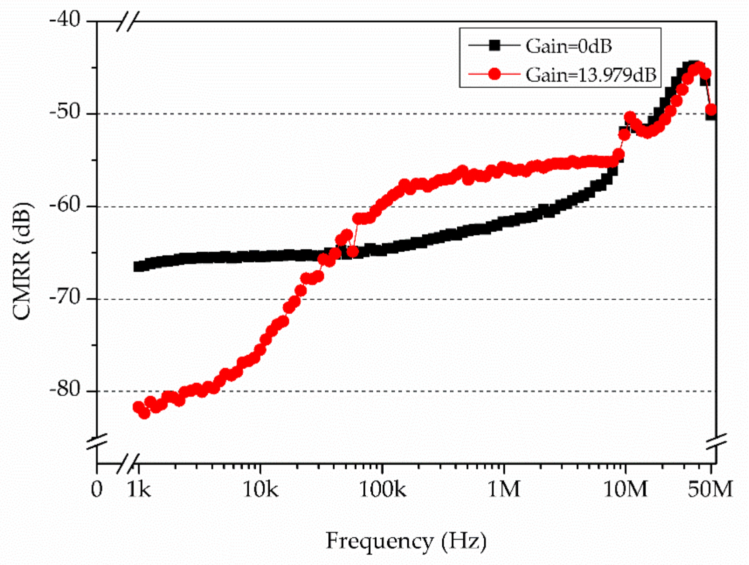

Supplementary Figure S2. The CMRR-frequency response of the preamplifier. The top bound of the CMRR can reach −80 dB when the gain is 13.979 dB. We used the HF2LI to measure the CMRR of the preamplifier. The HF2LI was set to AC coupling, 50 Ω input impedance, and differential mode. The signal output of the HF2LI generated a 1 Vpk sine wave which was used as the input signal of the preamplifier, and the output of the preamplifier was connected to the signal input 1 of HF2LI.

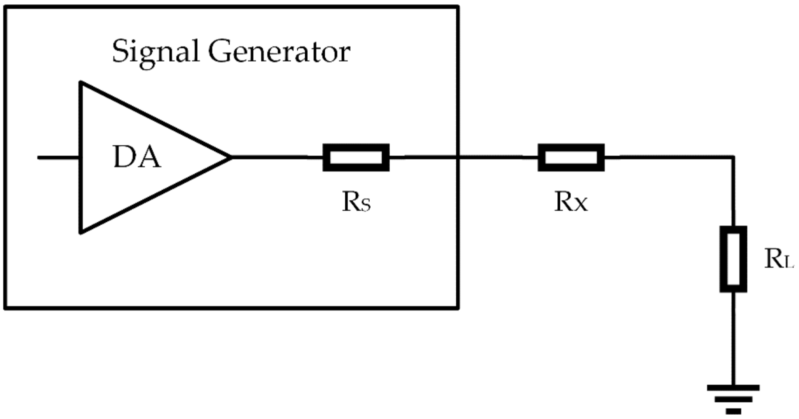

Supplementary Figure S3. The connection model of the input impedance measurement. The Rs is the impedance of the signal generator (commonly set to 50 Ω), Rx is the additional impedance and R_L is the input impedance of the preamplifier.

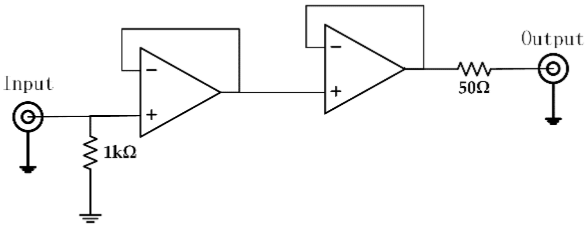

Supplementary Figure S4. The structure of the CA. The CA is implemented by two cascaded operational amplifiers (AD8065 by Analog Devices) with 1kΩ input impedance and 50 Ω output impedance.

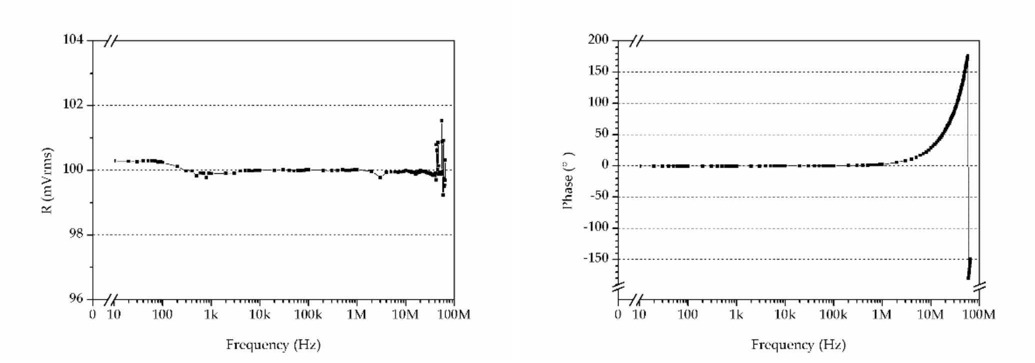

(a) (b)

Supplementary Figure S5. The frequency characteristics of the CA. We used the signal generator (Keysight 33622A) to generate a 100mVrms sine-wave as the input of the CA and measured the output of the CA by our DLIA.

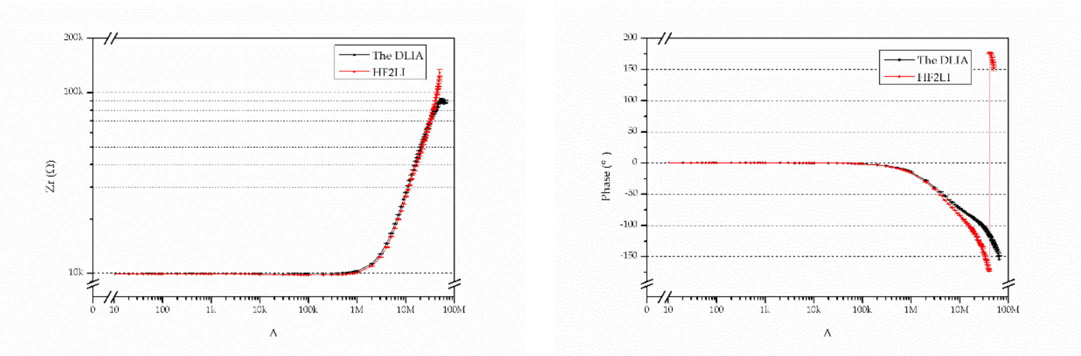

(a) (b)

Supplementary Figure S6. The measurement results of a 10 kΩ resistor by our DLIA and HF2LI. The increase of the measured impedance of the 10 kΩ resistor may be caused by parasitic effect.

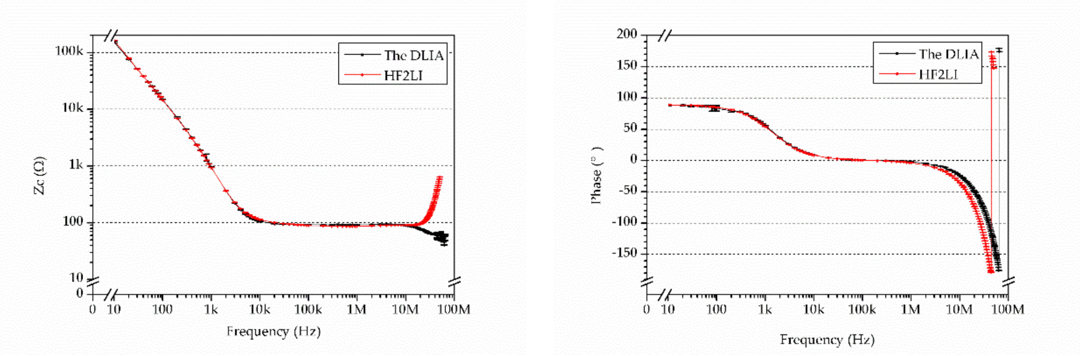

(a) (b)

Supplementary Figure S7. The measurement results of a 100 nF capacitor by our DLIA and HF2LI. The increase of the impedance measured by HF2LI when frequency exceeds 20 MHz is caused by the drop of the amplitude of output sine signal of the HF2LI (**Supplementary Figure S8**).

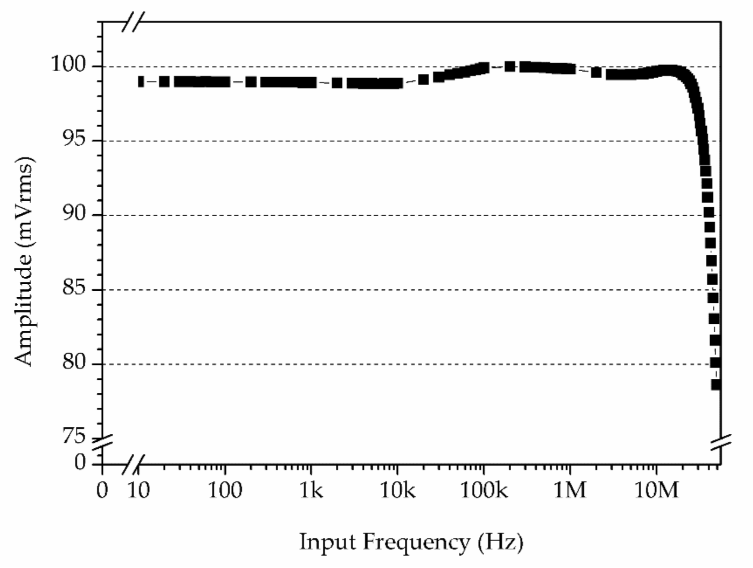

Supplementary Figure S8. The amplitude-frequency response of the HF2LI. We used the output sine signal of the HF2LI to generate a 100 mVrms signal and directly connect it to the input of the HF2LI. The amplitude is dropped when the frequency exceeds 20 MHz.

Supplementary Table S1. The comparison of the microfluidic impedance measurement between the DLIA and HF2IS.

	8 μm		10 μm	
	DLIA	HF2IS	DLIA	HF2IS
Standard Deviation (SD)	0.0026	0.0012	0.0032	0.0011
Coefficient of Variation (CV)	0.2703%	0.129%	0.3387%	0.128%
Mean A_r	0.9514	0.904	0.9384	0.885
Difference of Mean A_r of	DLIA		0.013	
8–μm and 10–μm Beads	HF2IS		0.019	

Supplementary Note S1. The calibration process in our DLIA.

We have used piecewise polynomial fitting to calibrate the output (both R and θ values) of the DLIA in ARM. The coefficients were calculated by the Matlab (by Mathworks, Inc., Natick, MA, USA) and then the output of the DLIA was calibrated in the ARM by the following Equation:

$$f(x_i) = p_{i_1} \cdot x_i^5 + p_{i_2} \cdot x_i^4 + p_{i_3} \cdot x_i^3 + p_{i_4} \cdot x_i^2 + p_{i_5} \cdot x_i + p_{i_6} \quad (1)$$

where the x_i is the output value without calibration in i[th] piece, $f(x_i)$ is the calibrated output value and p_i are the coefficients corresponding to the ith piece.

Supplementary Note S2. The input impedance measurement.

The connection model of the input impedance measurement is shown in **Supplementary Figure S3**. We calculated the input impedance by Equation 2:

$$\frac{V_{DA}}{R_L+(50+R_x)} \times R_L = V_{R_L} \quad (2)$$

By changing different R_X, we can get the R_L.

Here we set VDA to 1.5 Vpp (535 mVrms) at 1 Hz, R_x to 1 MΩ and 10 MΩ. The measured V_{R_L} is 485.5 mVrms and 267.9 mVrms correspondingly. The calculated R_L is 10.08 MΩ.
